# Vascular Factors in Patients with Midlife Sensorineural Hearing Loss and the Progression to Mild Cognitive Impairment

**DOI:** 10.3390/medicina59030481

**Published:** 2023-02-28

**Authors:** Valeria Del Vecchio, Laura Tricarico, Anna Pisani, Nicola Serra, Domenico D’Errico, Eugenio De Corso, Teresa Rea, Pasqualina M. Picciotti, Carla Laria, Giuseppe Manna, Annamaria Franzè, Rita Malesci, Anna Rita Fetoni

**Affiliations:** 1 Section of Audiology, Department of Neuroscience, Reproductive Sciences and Dentistry, University of Naples Federico II, Via Pansini, 5, 80131 Naples, Italy; 2 Otolaryngology Institute, Department of Head and Neck, Fondazione Policlinico Universitario A. Gemelli, IRCCS, 00168 Rome, Italy; 3 Department of Otolaryngology, Università Cattolica del Sacro Cuore, 00168 Rome, Italy; 4 Department of Public Health, University of Naples Federico II, Via Pansini, 5, 80138 Naples, Italy

**Keywords:** hearing loss, mild cognitive impairment, cognitive decline, anxiety, depression, midlife

## Abstract

*Background and Objectives*: Midlife hearing loss (HL) has been considered as a major modifiable risk factor for a later-life progression to dementia. Our aim was to detect a link between precocious sensorineural hearing loss (SNHL) and mild cognitive impairment (MCI) and their association to putative risk factors for a common pathology. *Materials and methods:* In this study, a retrospective case-control study was carried out. A total of 112 patients were enrolled as following: 81 patients with bilateral SNHL and 31 subjects with normal hearing, whose ages ranged from 50 to 65 years. Both groups performed pure tone audiometry, a tinnitus handicap inventory (THI), Mini-Mental State examination (MMSE), and the Montreal Cognitive Assessment (MoCA), Hospital Anxiety and Depression Scale (HADS-A and HADS-D). *Results:* The mean age was 58 ± 5.2 in SNHL patients and 53.2 ± 4.8 in the control group. The mean pure tone average in the SNHL group was 40.2 ± 18.7 dB HL on the right side and 41.2 ± 17.2 dB HL on the left side, while in the control group it was 12.5 ± 2.8 dB HL on right side and 12.4 ± 3.1 dB HL on left side. About 64% of patients with SNHL exhibited comorbidities, and the most common condition was hypertension. Altered MoCA test scores were significantly related to the pure tone averages in patients with SNHL compared to the control group (*p* = 0.0004), while the differences in the HADS-A and HADS-D were not significant. Furthermore, a significant correlation was observed in SNHL patients between an altered MoCA test and hypercholesterolemia (*p* = 0.043). *Conclusions:* Hearing impairment and screening tests to detect MCI should be considered in the midlife in order to carry out strategies to prevent the progression to dementia. Hypertension and hypercholesterolemia are two risk factors in the development of endothelial dysfunction, oxidative stress, and vascular inflammation, and may represent the common pathology linking the inner ear and brain damage.

## 1. Introduction

Sensorineural hearing loss (SNHL) is one of the major disabilities in adulthood and the prevalence of age-related hearing loss (ARHL) is rising with an increase in life expectancy. In fact, ARHL is the third most common disability in the elderly after hypertension and arthritis, with 360 million people suffering from a disabling form, corresponding to about 2/3 of subjects over 70 years of age and over 80% of those over 85 years old [1,2].

Nowadays, a growing interest in the literature has emerged on possible consequences of hearing loss (HL) due to longer life expectancy [3,4,5,6]. Based on the life-course model for the progression to health problems [7], HL has been considered as a modifiable risk factor for the later consequences of hearing deprivation on the quality of life, including depression, accelerated cognitive decline [8,9], and frailty [10] which is associated to poorer balance, falls, hospitalizations, and increased mortality [11].

Dementia is a syndrome leading to deterioration in cognitive function beyond what might be expected from the usual consequences of biological aging [12]. It affects memory, thinking, orientation, comprehension, calculation, learning capacity, language, and judgement and it is commonly accompanied, and occasionally preceded, by changes in mood, emotional control, behavior, or motivation. It is the result of a variety of diseases and injuries that primarily or secondarily affect the brain, such as Alzheimer’s disease which may contribute to 60–70% of cases [12]. The Worldwide Health Organization has declared that around 55 million people have dementia, with over 60% living in low- and middle-income countries. As the proportion of older people in the population is increasing in nearly every country, this number is expected to rise to 78 million in 2030 and 139 million in 2050 [12].

However, cognitive decline starts many years before the onset of dementia. Thus, a major goal is to detect the early stages of cognitive deterioration as mild cognitive impairment (MCI) characterized by isolated memory deficits with subjective memory problems, normal general cognitive functioning, and intact activities of daily living [13]. In fact, MCI is considered a prodromal stage that confers a high risk of developing dementia within 3 years [14,15]. Therefore, to improve diagnosis and prevention of MCI, which is often underestimated, is challenging.

On the way to potentially interrupt a progression to dementia, the early diagnosis of MCI is essential not only because these patients can still function at a fairly high level but because it is felt that interventions may be most effective in these early-stage patients [16]. On the other hand, among longer life modifiable risk factors, HL accounts for about 9% of dementia risk, a greater proportion than factors like hypertension, obesity, alcohol abuse, diabetes, and smoking [9]. The effect of the association of these modifiable predictors of the development of MCI and a conversion to dementia have rarely been studied.

It has also been suggested that MCI is associated with depression, itself a potentially modifiable risk factor for progression to dementia [17]. However, the association between midlife HL and risk of MCI is still understood and a few studies show conflicting results [18,19]. Consequently, the early detection of HL could promote its intervention and encourage the evaluation of other risk factors. Increasing experimental findings provide evidence for a common pathology linking the accelerated ARHL and memory defects and, subsequently, dementia [4,5,7,20,21]. According to the evidence that depression and anxiety are common in MCI [22], and are often diagnosed in the early stage of dementia, they typically manifest in patients with presbycusis and contribute to the progression of frailty [23]. Thus, symptoms of depression and anxiety are a spectrum of the most common and frequent comorbid manifestations present in patients with both presbycusis and dementia. Therefore, we aimed to evaluate whether, in patients affected by midlife SNHL, self-assessment questionnaires for depressive or anxiety profiles (i.e., HADS-A and HADS-D) and cognitive screening tools (i.e., MMSE and MoCA) could be effective in the detection of MCI. We also aimed to evaluate whether those with SNHL and positive tests for MCI expressed simple biomarkers of common pathology between inner ear and brain damage such as hypertension and hypercholesterolemia. Thus, in patients with precocious SNHL and MCI the progression to dementia could be prevented by counteracting biomarkers for a common pathology such as microvascular and metabolic risk factors.

## 2. Materials and Methods

This is a retrospective case-control study performed in audiology clinics at the Catholic University of the Sacred Heart of Rome, in the period between August and November 2022. Before enrolment, all patients received complete and comprehensible information about the tests to be administered and gave their written consent to their execution, according to the Ethical Committee recommendation as reported below.

The study was conducted on midlife patients, namely between 50 and 65 years with the ability to read, understand, and answer to the assigned questionnaires. The study group (SG) included patients with a diagnosis of bilateral SNHL and the control group (CG) included those with an ascertained normal hearing in the absence of subjective HL, tinnitus, and fullness.

The evaluation of all patients included an accurate investigation on the level of education, clinical history, and comorbidities.

Level of education is the progression from very elementary to more complicated learning experience. It was obtained by learning the highest finalized degree and categorized into five degrees: primary level, lower secondary level, upper secondary level, higher level (bachelor’s or equivalent level), and advanced level (master’s, doctorate, or equivalent level).

We excluded patients from both groups using the following criteria: previous diagnosis of HL, prior medical history of external injuries, middle ear disease, otosurgery, exposure to ototoxic drugs, family history of cognitive decline and/or neuropsychological disorders, neurodegenerative and cerebrovascular diseases, chronic heart and kidney diseases, and other health conditions that severely impair vision or mobility, as well as concomitant drug therapies with possible side effects on the central nervous system. The diagnosis of idiopathic HL was considered only after the exclusion of other possible causes, including a genetic evaluation and neuroimaging. Otherwise, in the absence of these evaluations, SNHL was defined as unknown.

The comorbidities achieved were common diseases reported in the population including hypertension, diabetes, hypothyroidism, hypercholesterolemia, and vestibular disorders. Hypertension was defined as having a mean systolic blood pressure of ≥ 130 mm Hg or a mean diastolic blood pressure of ≥80 mm Hg [24]. Diabetes was declared in cases of glycemia ≥126 mg/dL and hemoglobin A1c levels ≥ 6.5% [24]. Hypercholesterolemia was confirmed in individuals who had high-density lipoprotein levels ≤ 50 mg/dL and low-density lipoprotein levels ≥ 100 mg/dL [25]. Hypothyroidism was diagnosed in patients with laboratory data on TSH ≥ 4.5 mIU/mL 2.80 μIU/mL and f-T3 and f-T4 concentrations below or within normal range (f-T3 ≤ 4.2 pg/mL, f-T4 ≤ 15.5 pg/mL); an autoimmunity pattern was defined in the presence of serum antibody titers above the reference range (thyroglobulin antibodies TG-Ab ≥ 4.1 IU/mL and/or thyroid peroxidase antibodies TPO-Ab ≥ 5.6 IU/mL) [26].

Vestibular disorders were considered in subjects who had a previous diagnosis of peripheral vertigo as benign paroxysmal positional vertigo, endolymphatic hydrops, or vestibular neuritis.

### 2.1. ENT and Audiological Assessment

At the audiology clinics of the Catholic University of the Sacred Heart of Rome, we performed a complete audiological entry-level evaluation on all patients including the following: otoscopy, in order to determine the integrity of the conductive mechanism; tympanometry and acoustic reflex measurement (Grason Stadler Tympstar); and standard pure-tone audiometry in a double-walled, soundproof cabin (Amplaid 319 audiometer Amplaid Inc.) Air conduction hearing thresholds were measured at the range of frequencies from 0.125 kHz to 8 kHz with the use of headphones. Then, bone conduction hearing thresholds were measured for tonal stimuli at the range of frequencies from 0.25 to 4 kHz by bone conduction headphones. In the two groups, the pure tone average (PTA) was obtained by using the average from the thresholds at 0.5, 1, 2, and 4 kHz on a continuous scale, for each single side. Grades of hearing impairment used are consistent with the American Speech-Language-Hearing Association (ASHA) 2015 [27].

Normal hearing is indicated as 15 decibels in hearing level (dB HL) or better, slight HL as 16 to 25 dB HL, mild HL as 26 to 40 dB HL, moderate HL as 41 to 55 dB HL, moderately severe HL as 56 to 70 dB HL, severe HL as 71 to 90 dB HL, and profound HL as 91 dB HL or worse.

### 2.2. Tinnitus Handicap Inventory (THI)

Patients who reported the presence of tinnitus underwent an evaluation of tinnitus severity perception by the validated Italian version of the Tinnitus Handicap Inventory (THI) [28], an affordable and reliable tool used to evaluate the gravity of tinnitus subjective perception in heterogeneous populations. THI is a questionnaire composed of 25 items divided in three subgroups: the functional scale (11 items) concerns the discomfort of the patient in the cognitive, social, working, and physical areas; the emotional scale (9 items) includes a wide range of emotions generated by the tinnitus; and the last subgroup (5 items) reflects the catastrophic situation of the patient who cannot cope with this disorder, feel hopeless, and affirm that they are affected by a terrible disease. For each item, the patient can assign a value of 0, 2, or 4 points, depending on whether or not he fits himself in the described setting. The final score was reported according to the classification in five degrees: 1 slight (0–16 points), 2 mild (18–36 points), 3 moderate (38–56 points), 4 severe (58–76 points), and 5 catastrophic (78–100 points).

### 2.3. Cognitive Screening Test

Mini-Mental State Examination

A cognitive function screening was carried out by administering the Italian version of the Mini-Mental State Examination (MMSE) [29], consisting of a 30-point questionnaire to assess several mental abilities, including: short and long-term memory, attention span, concentration, language and communication skills, ability to plan, and ability to understand instructions. It requires about six to ten minutes to administer, although it may take longer depending on the extent of impairment. The MMSE has a high sensitivity for the identification of moderate and severe stages of dementia, but is of little use for the identification of early stages such as MCI [30]. This is because it is oriented to the evaluation of memory and language functions and does not consider executive functioning. If the screening test causes suspicion or more information is required, a complete neuropsychological evaluation is needed to ideally identify the patient’s specific deficits, differentiate between neurological and psychological aetiologies, Alzheimer’s dementia and other dementias, localize the deficits, and help formulate a personalized management plan [31]. The neurocognitive test was corrected for educational level and age intervals by standardizing the data with respect to this variable. For the MMSE, a result lower than 24 was considered pathological. Furthermore, the severity of cognitive impairment was distinguished by classifying the following scores: mild (19–23 points), moderate (10–18 points), and severe (≤9 points).

### 2.4. Montreal Cognitive Assessment (MoCA)

Neurocognitive screening was completed by the Montreal Cognitive Assessment (MoCA), a 10 min tool to assist first-line physicians in the detection of MCI. The Italian version [32] is a one-page 30-point test exploring six different domains, as follows: memory recall, visuospatial construction, executive functions, attention-concentration and working memory, language, and finally orientation to time and place.

The short-term memory recall task (5 points) involves two learning trials of five nouns and delayed recall after approximately 5 min. Visuospatial abilities are assessed using a clock-drawing task (3 points) and a three-dimensional cube copy (1 point). Multiple aspects of executive functions are assessed using the Trail Making B task (1 point), a phonemic fluency task (1 point), and a two-item verbal abstraction task (2 points). Attention, concentration, and working memory are evaluated using a sustained attention task (target detection using tapping; 1 point), a serial subtraction task (3 points), and a forward and backward digits task (1 point each). Language is assessed using a three-item confrontation naming task with low-familiarity animals (lion, camel, rhinoceros; 3 points), repetition of two syntactically complex sentences (2 points), and the aforementioned fluency task. Finally, orientation to time and place is evaluated (6 points). Compared to the similar MMSE test, the MoCA test has more words to remember (5 vs. 3), fewer learning tests (2 vs. 6), and more time between immediate recall and delayed recall (5 min vs. 2 min), this highlights the superior sensitivity of the MoCA for detecting amnesic MCI compared to the MMSE. The evaluation has scores from 0 to 30 points: the highest score reflects a high function, but if the subject has more than 12 years of education, one point is subtracted to correct the test. A result equal to or greater than 26 indicates a condition of normality [33].

### 2.5. Psychological Distress Screening Test: The Hospital Anxiety and Depression Scale (HADS)

The prevalence of emotional disorders was evaluated by the validated Italian version of Hospital Anxiety and Depression Scale (HADS) [34], a self-report questionnaire designed to screen anxious (subscale HADS-A) and depressive states (subscale HADS-D) in patients with various chronic conditions, in non-psychiatric settings [35] with the aim of detecting any symptoms and trying a psychological therapeutic approach. Each subscale consists of seven items with a 4-point ordinal response format (ranging from 0 to 3). The subscale HADS-A investigates the presence of states of tension, nervousness, fear, worries, inability to relax, restlessness, and panic attacks. The subscale HADS-D analyses the depression symptoms with items on low mood, sense of slowdown, anhedonia, loss of interest in body care, and pessimism. Scores ranges from 0 to 21 in each subscale, and the severity was distinguished by stratifying the symptoms during the past week in the following ranges: normal (0–7 points), mild (8–10 points), moderate (11–15 points), and severe (16–21 points).

### 2.6. Ethical Consideration

Before enrolment, all patients received complete and comprehensible information about the tests administered and informed consent was signed by all participants in the study. Anonymity was guaranteed for all participants. No economic incentives were offered or provided for participation in this study. The study was performed under the ethical considerations of the Helsinki Declaration. The study was approved by the Local Ethics Committee under protocol code 0025860/22 on 3 August 2022.

### 2.7. Statistical Analysis

Data are presented as number and percentage for categorical variables and continuous data are expressed as mean ± standard deviation (SD), unless otherwise specified.

The test for normal distribution was performed by Shapiro-Wilk test. The chi-square test was used to evaluate significant differences between the two groups. The Fisher’s exact test was used where the chi-square test was not appropriate. A binomial test was performed to compare two mutually exclusive proportions or percentages in groups. In order to analyze the differences among three or more modalities of a variable, the chi-square goodness of fit was used. To obtain the type of comorbidity variable, a multiple comparison Cochran’s Q test was used to compare the differences among percentages under the consideration of the null hypothesis that there were no differences between the variables. When the Cochran’s Q test was positive (*p*-value < 0.05), then a minimum required difference for a significant difference between two proportions was calculated using the minimum required differences method (MRD) with the Bonferroni *p*-value corrected for multiple comparisons according to Sheskin [36].

A *t*-test was used to compare the mean of unpaired samples. When the distribution of samples was not normal, the Mann-Whitney test was used.

Finally, the degree of the relationship between the better ear PTA and MoCA was computed using Spearman’s correlation coefficient rho.

We considered all statistical tests with *p*-value < 0.05 to be significant. All data were analyzed with Matlab statistical toolbox version 2008 (MathWorks, Natick, MA, USA) for 32-bit Windows.

## 3. Results

The study included 112 subjects, 81 in SG and 31 in CG. Namely, we enrolled 61 (75.3%) males and 20 (24.7%) females with a mean age of 58 ± 5.2 years in SG and 20 (64.5%) males and 11 (35.6%) females with a mean age of 53.2 ± 4.8 years in CG with a significant difference for age among the two groups. Demographic features and educational levels are reported in Table 1.

The pure tone audiometry showed bilateral SNHL in the SG and absence of HL in CG (Table 2).

The mean value of PTA was 40.2 ± 18.7 dB HL on the right side and 41.2 ± 17.2 dB HL on the left side in the SG and 12.5 ± 2.8 dB HL on the right side and 12.4 ± 3.1 dB HL on the left side in the CG. Indeed, the mean value of better ear PTA was 36.6± 14.6 dB HL in the SG and 12 ± 3.1 dB HL in the CG.

The etiology of SNHL is shown in Table 3, it is unknown in 35 (43.2%), idiopathic in 3 (3.7%), related to noise-induced hearing loss in 35 (43.2%), endolymphatic hydrops in 4 (4.9%), cochlear otosclerosis in 2 (2.5%), and temporal bone fracture in 2 (2.5%).

In all patients, the presence of common comorbidities was investigated. A significant difference was found for the overall presence of comorbidities in the SG as compared to the CG (64.2% in SG vs. 35.5% in CG, *p* = 0.0061), but no significant difference was identified in number (*p* = 0.082) of associated morbidity and type (*p* = 0.78) among the two groups (Table 3). Namely, in the SG we found that one morbidity was present in 31 (38.3%) patients and two comorbidities in 15 (18.5%) patients, while only few patients were affected by three (4 patients, 4.9%) or four (2 patients, 2.5%) comorbidities. Interestingly, among comorbidities there was an overall significant difference in the SG (*p* = 0.001). Hypertension was more significantly reported in the SG (33.3%, *p* < 0.0001). All data are detailed in Table 3.

Furthermore, the association of HL to tinnitus was evaluated. A total of 48/81 (59.3%) of patients in the SG experienced a continuous tinnitus. There was a significant presence of subjects with bilateral tinnitus (34/48, corresponding to 70.8%, *p* < 0.0001) that was unilateral in 14/48 (29.1%). The average score obtained by THI was 40.6 ± 26.4 and the degree of discomfort was not severe in 35 (72.9%) patients. Namely, tinnitus-induced discomfort was slight for 11/48 (22.9%), mild for 12/48 (25%), moderate for 12/48 (25%), severe for 7/48 (14.6%), and catastrophic for 6/48 (12.5%).

All enrolled subjects were then evaluated for their cognitive levels as reported in Table 4 by using the MMSE and MoCA screening tests. The median score of MMSE in the SG was 26 (24.15, 27.2), while it was 27.2 (25.2, 27.85) in the CG. Therefore, no significant difference was observed among the two groups as detailed in Table 5.

The table describes the results of MMSE, MoCA, and HADS for evaluation of anxiety (HADS–A) and depression (HADS-D). It reports the mean, the median, the percentage (%), and the number (*n*) of subjects in the total group (TG), control group (CG), and study group (SG). SD = standard deviation, IQR = interquartile range, * = significant test, ** = significant more frequent, Cg = chi-square goodness fit with post hoc z-test, MW = Mann-Whitney test, C = chi-square test, F = Fisher’s exact test.

Conversely, the median score of MoCA was 24 (23, 26) in the SG and 27 (25.75, 28) in the CG with a significant difference among the two groups (*p* = 0.0006), while lower MoCA test scores were observed in the SG as compared to the CG (64.4% vs. 24.1%, respectively, *p* = 0.0004).

Furthermore, additional analyses were performed in order to detect possible correlations in each group between MoCA test scores and comorbidities: hypertension, diabetes, hypothyroidism, hypercholesterolemia, and vestibular disorders. Table 5 shows no significant correlations in the CG. Remarkably, in the SG, we found a significant relationship between altered MoCA test scores and hypercholesterolemia (*p*= 0.043), as reported in Table 6.

We evaluated neuropsychological distress by using HADS-A and HADS-D subscales that revealed no significant difference between the SG and the CG for the score (HADS-A median: 6 (4, 9.75) in SG vs. 5 (1.75, 7,25) in CG, *p* = 0.10, HADS-D median: 4 (1.25, 8) in SG vs. 2 (0.75, 5.25) in CG, *p* = 0.06 and for severity of symptoms (*p* = 0.64 and *p* = 0.26)). In detail, for HADS-A, the SG showed normal results in more than half of the subjects (68.7%,), nevertheless scores related to mild, moderate, and severe symptoms of anxiety were noted as follows: mild in 13.4%, moderate in 13.4%, and severe in 4.5% of patients). Additionally, in the CG the absence of anxiety was detected more often (76.5%), while in this group altered scores for mild (17.6%) and moderate (5.9%) symptoms were noted. The overall differences between the two groups were not significant. Similarly, normal scores for HADS-D were evaluated in 73.1% of patients in the SG, while test values for mild, moderate, and severe symptoms of depression were found in 9.0%, 16.4%, and 1.5% of patients, respectively. In the CG, only a few patients presented mild altered questionnaires (5.9%). Even for the depressive profiles, no differences were found between the SG and the CG. Taken together, about 64% of patients with SNHL exhibited one or more comorbidities, and the most common condition was hypertension. The MMSE was unable to discern the increased risk for MCI between normal hearing and patients with SNHL. Remarkably, altered MoCA test scores were significantly related to patients with SNHL compared to normal hearing subjects, while the differences in the HADS-A and HADS-D were not significant. Finally, a significant correlation was observed in individuals with SNHL between altered MoCA test and hypercholesterolemia.

Finally, we performed a correlation analysis between the better ear PTA and MoCA using Spearman’s correlation coefficient rho. For the CG and SG groups, no association between the better ear PTA and MoCA were observed(rho = 0.02, *p* = 0.91; rho = −0.24, *p* = 0.07, respectively).

## 4. Discussion

Hearing impairment in the elderly has been considered in relation to senescence, however, its prevalence and consequences on the cognitive function are still controversial. Increased experimental and clinical findings provide evidence for a relationship between HL and cognitive decline, indicating its onset in the midlife as a major modifiable risk factor for later progression to dementia. Thus, considering that HL is more easily diagnosed in adulthood, we investigated if the concomitant use of simple and reliable screening tests for detection of depression, anxiety, and MCI, as prodromal to later-life severe cognitive decline, could be helpful to identify subjects with an increased risk for developing dementia. Herein, neurocognitive assessment with MoCA test revealed lower scores in more than half of the patients with SNHL (38/59–64,4%; *p* = 0.0004).

Accordingly, MMSE scores were worse in subjects with SNHL, even if the difference was not statistically significant. Nevertheless, although it is the most commonly used scale in cognitive function evaluation, it is better used for elderly people [37]. Indeed, previous studies confirmed that the sensitivity of MoCA in detecting MCI was 90% superior to the sensitivity of MMSE (18%) because MoCA test checks executive functions, visuospatial thinking, working memory, memory recall, concentration/attention, language, and orientation [38,39]. Therefore, it focuses on many domains not analyzed by MMSE like executive functions, higher-level language abilities, and complex visuospatial processing. The MoCA test better meets the criteria for screening tests in order to predict the development of MCI prodromal to cognitive decline [37].

Furthermore, patients exhibited a good compliance in performing neurocognitive evaluation, although we did not use an adapted version of the MoCA test for hearing-impaired subjects. In fact, the severity of HL was mild to moderate in about 75% of the SG patients. However, the PTA scores are not completely representative of the hearing levels, considering that we found sloping shape curves for the higher frequencies in almost all patients. On the other hand, this condition was favorable for the interaction between examiner and patient, and hearing difficulties did not invalidate the execution of neurocognitive tests. Thus, our data suggest that in patients with mild to moderate HL, the MoCA test can be helpful for early detection of individuals who could be at risk for cognitive dysfunction, and might have impact on the follow up of these patients and on the rehabilitative potential.

Despite the fact that it is widely accepted that HL is an independent modifiable risk factor for dementia [8,9], its impact in the midlife as a potential marker for the later-life consequences on cognition is still poorly understood.

Nevertheless, the causative link between ARHL and the development of cognitive decline has not previously been clarified. Traditionally, based on the Schuknecht classification [40], cochlear damage was considered to be the main cause of ARHL due to outer hair cell loss (sensory presbycusis), primary degeneration of spiral ganglion cell (neural presbycusis), atrophy of stria vascularis (strial presbycusis) and, more rarely, stiffness of the basilar membrane (mechanical cochlear/conductive hypothetical presbycusis) [40]. On the other hand, Schuknecht topology does not include the auditory perceptual dysfunction, which cannot be explained on the basis of peripheral HL and refers to impairment in central auditory pathways. These are involved in central auditory processing disorders and may explain the decline of discrimination, particularly in noisy environments [5]. Therefore, Gates and Mills have classified ARHL in peripheral and central presbycusis, considering HL as a multifactorial complex disorder with both genetic susceptibility and environmental factors such as noise and ototoxic drugs, which contribute to the early onset of SNHL and to the involvement of central auditory pathways [41].

Thus, several mechanistic hypotheses have been proposed to explain the link between hearing impairment and cognitive decline: cognitive load on perception, degradation of audiological information, sensory deprivation, and common causes [5,42].

The cognitive load on perception hypothesis declares that a cognitive impairment can decrease the resources available for auditory perception, manifesting as HL, and it can reduce the understanding of speech. Then, degradation of audiological information suggests that hearing impairment causes a degradation of inputs to the brain, leading to an abnormal stimulation with changes and functional modification in the auditory pathway and central nervous system resulting in the development of dementia. The direct effect of auditory deprivation seems to be the atrophy of the primary auditory cortex and temporal lobe [5]. Moreover, a common etiopathogenesis is assumed in cochlear damage and microvascular cortical neurodegeneration is frequently observed in subjects with cognitive decline. Vascular risk factors as diabetes, atherosclerosis, and hypertension, but also noise exposition and ototoxic drugs, such as cisplatin and aminoglycosides, are known to induce oxidative stress and consequently the degeneration of stria vascularis, sensorineural epithelium, and neurons in the spiral ganglion and auditory cortex of the central auditory pathways [5]. Therefore, focusing on common comorbidities in our sample, these were present in 64.2% of patients with SNHL and hypertension was the disease more frequently reported in the SG (33.3%, *p* < 0.0001). Interestingly, we found a correlation between worse results in the MoCA test and hypercholesterolemia.

In previous studies in the experimental model, an oedema in stria vascularis was found [43,44] in mice with hypercholesterolemia [45]. Nevertheless, although stria vascularis is a well-known site of damage in systemic diseases, the actual underlying pathogenesis is still unknown [46]. We previously studied common markers shared between HL and ARHL in an experimental model, demonstrating that noise-induced hearing loss can worsen/accelerate redox status imbalance including the increase of reactive oxygen species production; lipid peroxidation; dysregulation of endogenous antioxidant response, vascular dysfunction, such as increased expression of hypoxia-inducible factor-1alpha and vascular endothelial growth factor in the cochlea, both of which are involved in the dysregulation of redox status and inflammatory pathway activation [3]. Moreover, a recent study suggests that hypercholesterolemia aggravates sevoflurane-induced cognitive impairment in aged rats by inducing neurological inflammation and apoptosis [47].

However, a wide sample of patients may better explain the correlation between HL and other systemic diseases such as diabetes. This is likely because, in our study, all patients were recruited without previous diagnosis of HL, patients with chronic metabolic diseases more frequently undergo audiological testing.

We also evaluated the presence of tinnitus, that has been studied as an early auditory symptom, and the emotional effects which could accelerate a cognitive decline. Among the cases, 48/81 (59.3%) patients declared they suffered from it, more often bilaterally (34/48-70.8%) and not a severe grade (35/48–72.9%).

Previous studies assert an increased risk of psychiatric comorbidities among patients with tinnitus [48], but they also report mild psychiatric symptoms and a worsening of quality of life [49,50]. Recent literature argues there is a complex association among tinnitus perception, emotional disorders, and cognitive dysfunction, however the causal link is still not fully understood [51,52]. Tinnitus may be a trigger of neuropsychiatric distress, or it may prompt a subclinical condition, but the reverse association is also supposed in which depression activates an exacerbation of the symptom.

Furthermore, the impact of tinnitus on emotional well-being may cause a cognitive load leading to failures in daily activities and in executive control tasks, in accordance with the so-called “load theory” [52,53]

Although it is largely demonstrated that psychiatric discomfort is present in a large number of tinnitus suffers [23,48], actual evidence does not allow us to establish, with certainty, if tinnitus matters as an independent risk factor for cognitive impairment or the evolution to dementia [49]. Nevertheless, a previous evaluation of glucose metabolic connectivity through FDG-PET has already shown a lower metabolism in the right superior temporal pole and in the fusiform gyrus among subjects with MCI and tinnitus, unlike those with only a cognitive impairment [54]. In addition, a lower grey matter volume in the right insula was detected among patients with a worse THI score, suggesting a correlation between the alteration of the brain and the emotional reaction to tinnitus. Therefore, the pathophysiology of tinnitus and the central neural changes that it determines might explain the relationship between the impact of tinnitus and the development of cognitive decline [49]. It has been previously demonstrated that the long-term hearing deprivation of auditory inputs can impact cognitive performance by decreasing the quality of communication, leading to social isolation and depression and facilitating dementia [3,4,5]. Our results from HADS-A and HADS-D showed traits of neuropsychological disorders in the SG more than controls, namely depression, although the difference was not significant. Accordingly to a recent systematic review, HL has been associated with increased risk for psychological distress and it reduces quality of life, which also seems to improve after the fitting of hearing aids [6]. It is likely that in our sample the association with tinnitus was not relevant, however this point will be better clarified by increasing the study sample.

On the other hand, HL as well as tinnitus have been linked to structural changes within the central nervous system [55], but also in other structures involved in high-level cognitive control functions such as the dorsolateral prefrontal cortex and anterior cingulate cortex [56]. Moreover, hearing impairment is clearly related to a brain remodeling [56], which compensates for the loss through the activation of collateral pathways. It requires more cognitive resources leading to mental fatigue. This is in accordance with worse performances detected by the MoCA test in our own as well as previous studies, which explains more difficulties in attentive and executive functions in patients with HL even if they underwent hearing rehabilitation through the use of hearing aids or cochlear implantation [57].

In addition, a recent study of Alzheimer’s disease using a mouse model has demonstrated that noise exposure induced midlife persistent synaptic and morphological alterations in the auditory cortex associated with earlier hippocampal dysfunction, indicating anticipated memory deficits compared to the expected time-course of the neurodegeneration, as well as signs of molecular damage, including increased tau phosphorylation, neuroinflammation, and redox imbalance [20]. Despite progress in pre-clinical models, there is no evidence, and clinicians lack sensitive non-invasive biomarkers for HL and its consequences. Our findings are in line with the aim of a personalized approach to HL by detecting indicators for the disease, especially in the midlife which is an effective period for rehabilitation.

The major limitations of our study are the age and gender distribution [58,59] of the two groups because the SG is older than the CG. Unfortunately, the recruitment of subjects with normal hearing function and absence of other exclusion criteria is difficult during midlife because most of the patients come to the outpatient clinic only in the presence of symptoms. Moreover, even if they declare normal hearing function it is common to detect subjects with a sensorineural dip on high frequencies at pure tone audiometry.

Our results support the evidence that hearing impairment can be considered an effective risk factor for cognitive decline such as the early onset of MCI and demonstrate that the role of prevention or early detection of HL, as well as encouraging an intervention program. Moreover, in patients undergoing audiological evaluation, a multidisciplinary approach may offer a global evaluation that also uses questionnaires, which permit a prompt detection of many underestimated clinical cases that require more investigation, and to carry out strategies to prevent the evolution towards dementia. Questionnaires can be useful in addressing a clinical approach for identifying the subjects with risk factors for cognitive decline (i.e., MCI) during midlife, and eventually paired with prevention programs for comorbidities and hearing rehabilitation. Several studies have already declared the importance of auditory rehabilitation, which may improve both cognitive decline [60] and neuropsychological disorders [61,62]. Unfortunately, validating the biomarkers of oxidative stress or inflammation indicating inner ear stress are ineffective [63], thus hypertension and hypercholesterolemia, which are two risk factors in the development of endothelial dysfunction, oxidative stress, and vascular inflammation, may represent conditions for a common pathology linking inner ear and brain damage.

## 5. Conclusions

A screening evaluation for MCI should be always performed in patients with SNHL in midlife. It should be also considered in the co-presence of vascular factors, such as hypertension and hypercholesterolemia which are two risk factors in the development of endothelial dysfunction, oxidative stress, and vascular inflammation and may represent the common pathology linking inner ear and brain damage.

An early detection and intervention program for MCI in midlife may be crucial to counteract cognitive decline due to the aging of society, with increased life expectancies, in order to avoid health and economic costs.

## Figures and Tables

**Table 1:** Demographic features and educational levels in the study group and control group.

Variables	Total Group	Control Group	Study Group
Subjects	112	31	81
Age y.o.			
Mean (SD)	56.7 (5.5)	53.2 (4.8)	58.0 (5.2)
Median (IQR)	57 (50, 61.25)	50 (50, 55.75)	59 (53, 62)
Gender %(*n*)			
Males	72.3 (81)	64.5 (20)	75.3 (61)
Females	27.7 (31)	35.6 (11)	24.7 (20)
Education level %(*n*)			
Lower Secondary	31.3 (35)	29.0 (9)	32.1 (26)
Upper Secondary	49.1 (55)	41.9 (13)	51.9 (42)
Higher	13.4 (15)	29.0 (9)	7.4 (6)

The table describes the percentage (%) and the number (*n*) of subjects in the total group (TG), control group (CG), and study group (SG), the mean with standard deviation (SD), as well as the median age with interquartile range (IQR) in years old (y.o.), the gender (males, females), and the education level (secondary level, upper secondary level, higher level (bachelor’s or equivalent level).

**Table 2:** Hearing threshold in the study group and control group.

Variables	Total Group	Control Group	Study Group	CG vs. SG *p*-Value (Test)
Right PTA				
Mean (SD)	32.5 (20.2)	12.5 (2.8)	40.2 (18.7)	
Median (IQR)	28.1 (15, 37.5)	15 (10, 15)	31.25 (27.5, 45.3)	
Severity %(*n*)				
Normal	27.7 (31)	100 (31) ** (Cg)	0.0 (0)	
Slight	0.0 (0)	0.0 (0)	0.0 (0)	
Mild	51.8 (58)	0.0 (0)	71.6 (58) ** (Cg)	*p* < 0.0001 (F)
Moderate	8.0 (9)	0.0 (0)	11.1 (9)	CG Normal *,
Moderate-Severe	5.4 (6)	0.0 (0)	7.4 (6)	*p* < 0.001 (Z)
Severe	5.4 (6)	0.0 (0)	7.4 (6)	
Profound	1.8 (2)	0.0 (0)	2.5 (2)	
Left PTA				
Mean (SD)	33.3 (19.6)	12.4 (3.1)	41.2 (17.2)	
Median (IQR)	30 (15, 40.3)	15 (10, 15)	33.75 (28.75, 50)	
Severity %(*n*)				
Normal	27.7 (31)	100 (31) ** (Cg)	0.0 (0)	
Slight	0.0 (0)	0.0 (0)	0.0 (0)	
Mild	47.3 (53)	0.0 (0)	65.4 (53) ** (Cg)	*p* < 0.0001 (F)
Moderate	13.4 (15)	0.0 (0)	18.5 (15)	CG Normal *,
Moderate-Severe	8.0 (9)	0.0 (0)	11.1 (9)	*p* < 0.0001 (Z)
Severe	2.7 (3)	0.0 (0)	3.7 (3)	
Profound	0.9 (1)	0.0 (0)	1.2 (1)	
Better ear PTA				
Mean (SD)	29.8 (16.7)	12.0 (3.1)	36.6 (14.6)	
Median (IQR)	27.5 (15, 33.75)	10 (10, 15)	30 (27.5, 38.75)	
Severity %(*n*)				
Normal	27.7 (31)	100 (31) ** (Cg)	0.0 (0)	
Slight	0.0 (0)	0.0 (0)	0.0 (0)	
Mild	56.3 (63)	0.0 (0)	77.8 (63) ** (Cg)	*p* < 0.0001 (F)
Moderate	8.0 (9)	0.0 (0)	11.1 (9)	CG Normal *,
Moderate-Severe	5.4 (6)	0.0 (0)	7.4 (6)	*p* < 0.0001 (Z)
Severe	1.1 (2)	0.0 (0)	2.5 (2)	
Profound	0.9 (1)	0.0 (0)	1.2 (1)	

The table describes the mean and the median of pure tone average (PTA) in each side (right, left) and in the better ear in the total group (TG), control group (CG), and study group (SG). The threshold of hearing function is classified following the ASHA 2015 [27]: normal hearing (≤15 dB HL), slight (16–25 dB HL), mild (26–40 dB HL), moderate (41–55 dB HL), moderately severe (56–70 dB HL), severe (71–90 dB HL), and profound (≥ 91 dB HL). SD = standard deviation, IQR = Interquartile range, * = significant test, ** = significant more frequent, Cg = chi-square goodness fit with post hoc z-test, MW = Mann-Whitney test, F = Fisher’s exact test, Z = z-test.

**Table 3:** The comorbidities in the study group and control group.

Variables	Total Group %(*n*)	Control Group %(*n*)	Study Group %(*n*)	CG vs. SG *p*-Value (Test)
Comorbidity	56.3 (63)	35.5 (11)	64.2 (52)	0.0061 * (C)
Nr = 0	43.8 (49)	64.5 (20) ** (Cg)	35.8 (29) ** (Cg)	
Nr = 1	34.8 (39)	25.8 (8)	38.3 (31) ** (Cg)	
Nr = 2	15.2 (17)	6.5 (2)	18.5 (15)	
Nr = 3	4.5 (5)	3.2 (1)	4.9 (4)	
Nr = 4	1.8 (2)	0.0 (0)	2.5 (2)	0.084 (F)
Type of comorbidity				
hypertension	27.7 (31)	12.9 (4)	33.3 (27) ** (Q)	
diabetes	6.3 (7)	3.2 (1)	7.4 (6)	
hypothyroidism	9.8 (11)	6.5 (2)	11.1 (9)	
hypercholesterolemia	10.7 (12)	3.2 (1)	12.4 (10)	0.81 (F)

The table describes the percentage (%) and the number (*n*) of subjects in the total group (TG), control (CG), and study group (SG) with an associated comorbidity, detailing the number (Nr) and the type of them. * = significant test, ** = significant more frequent, Cg = chi-square goodness fit with post hoc z-test, C = chi-square test, F = Fisher’s exact test, Q = Cochran’s Q test and MRD = minimum required differences method with Bonferroni *p*-value corrected post hoc test.

**Table 4:** The cognitive and neuropsychological evaluation in the study group and control group.

Variables	Total Group	Control Group	Study Group	CG vs. SG *p*-Value (Test)
MMSE				
Mean (SD)	25.8 (2.2)	26.5 (2.3)	25.6 (2.2)	
Median (IQR)	26.2 (24.2, 27.2)	27.2 (25.2, 27.85)	26 (24.15, 27.2)	0.06 (MW)
Severity %(*n*)				
Normal	86.6 (71/82)	94.1 (16/17) ** (Cg)	84.6 (55/65) ** (Cg)	
Mild	13.4 (11/82)	5.9 (1/17)	15.4 (10/65)	
Moderate	0.0 (0/82)	0.0 (0/17)	0.0 (0/65)	
Severe	0.0 (0/82)	0.0 (0/17)	0.0 (0/65)	0.45 (F)
MoCA				
Mean (SD)	25.2 (2.7)	26.5 (2.9)	24.6 (2.4)	
Median (IQR)	25 (23.75, 27)	27 (25.75, 28)	24 (23, 26)	0.0006 * (MW)
Severity %(*n*)				
Normal	48.9 (43/88)	75.9 (22/29) ** (Cg)	35.6 (21/59)	
Altered	51.1 (45/88)	24.1 (7/29)	64.4 (38/59) ** (Cg)	0.0004 * (C)
*HADS–A*				
Mean (SD)	6.4 (4.4)	4.6 (3.4)	6.8 (4.5)	
Median (IQR)	6 (3, 9)	5 (1.75, 7.25)	6 (4, 9.75)	0.10 (MW)
Severity %(*n*)				
Normal	70.2 (59/84)	76.5 (13/17) ** (Cg)	68.7 (46/67) ** (Cg)	
Mild	14.3 (12/84)	17.6 (3/17)	13.4 (9/67)	
Moderate	11.9 (10/84)	5.9 (1/17)	13.4 (9/67)	
Severe	3.6 (3/84)	0.0 (0/17)	4.5 (3/67)	0.87 (F)
*HADS–D*				
Mean (SD)	4.8 (4.4)	2.8 (2.8)	5.3 (4.6)	
Median (IQR)	3 (1, 7)	2 (0.75, 5.25)	4 (1.25, 8)	0.06 (MW)
Severity %(*n*)				
Normal	77.4 (65/84)	94.1 (16/17) ** (Cg)	73.1 (49/67) ** (Cg)	
Mild	8.3 (7/84)	5.9 (1/17)	9.0 (6/67)	
Moderate	13.1 (11/84)	0.0 (0/17)	16.4 (11/67)	
Severe	1.2 (1/84)	0.0 (0/17)	1.5 (1/67)	0.23 (F)

**Table 5:** Relationship analysis between the better ear PTA and MoCA parameter with comorbidities for the control group.

CG Group			
**Parameters**	**MoCA (*n* = 22)** **% Normal Score**	**MoCA (*n* = 7)** **% Altered Score**	***p*-Value (Test)**
Diabetes	21 (no), 1 (yes)	7 (no), 0 (yes)	1.0 (F)
Hypothyroidism	21 (no), 1 (yes)	6 (no), 1 (yes)	0.39 (F)
Hypercholesterolemia	21 (no), 1 (yes)	7 (no), 0 (yes)	1.0 (F)
Hypertension	17 (no), 5 (yes)	6 (no), 1 (yes)	1.0 (F)
Vestibular disorders	21 (no), 1 (yes)	7 (no), 0 (yes)	1.0 (F)
**Parameters**	**Better Ear PTA** **Yes Comorbidity**	**Better Ear PTA** **No Comorbidity**	***p*-Value (Test)**
Diabetes	10.0	12.1 ± 3.1	No performed test
Hypothyroidism	11.34 ± 1.8	12.1 ± 3.1	No performed test
Hypercholesterolemia	10.0	12.1 ± 3.1	No performed test
Hypertension	12.5 (10, 15)	10 (10, 15)	0.79 (MW)
Vestibular disorders	-	12.0 ± 3.1	No performed test

The table describes the relationship analysis between the better ear PTA and MoCA parameter with comorbidities for the control group. MoCA test is considered altered with a score less than 26. F = Fisher’s exact test, MW = Mann-Whitney test; no = no presence, yes = presence.

**Table 6:** Relationship analysis between the better ear PTA and MoCA parameter with comorbidities for the study group.

SG Group			
**Parameters**	**MOCA (*n* = 21)** **% Normal Score**	**MOCA (*n* = 38)** **% Altered Score**	***p*-Value (Test)**
Diabetes	20 (no), 1 (yes)	35 (no), 3 (yes)	1.0 (F)
Hypothyroidism	19 (no), 2 (yes)	33 (no), 5 (yes)	1.0 (F)
Hypercholesterolemia	21 (no), 0 (yes)	31 (no), 7 (yes)	0.043 * (F)
Hypertension	15 (no), 6 (yes)	23 (no), 15 (yes)	0.40 (C)
Vestibular disorders	21 (no), 0 (yes)	32 (no), 6 (yes)	0.08 (F)
**Parameters**	**Better Ear PTA** **Yes Comorbidity**	**Better Ear PTA** **No Comorbidity**	***p*-Value (Test)**
Diabetes	35 (27.5, 38.8)	30 (27.5, 38.8)	0.43 (MW)
Hypothyroidism	40 (26.3, 46.3)	30 (27.5, 38.1)	0.41 (MW)
Hypercholesterolemia	40 (27.5, 39.7)	30.6 (27.5, 37.5)	0.97 (MW)
Hypertension	32.5 (26.7, 48.1)	30 (27.5, 37.5)	0.39 (MW)
Vestibular disorders	30 (27.2, 32.2)	30.6 (27.5, 39.4)	0.37 (MW)

The table describes the relationship analysis between the better ear PTA and MoCA parameter with comorbidities for the study group. MoCA test is considered altered with a score less than 26. * significant test (*p* < 0.05), C = chi square test, F = Fisher’s exact test, MW = Mann-Whitney test; no = no presence, yes = presence.

## Data Availability

Data available on request.
